# Post-pandemic surge of *Mycoplasma pneumoniae* in Ontario, 2024: molecular surveillance and resistance trends relative to 2018–2023

**DOI:** 10.1128/jcm.00188-26

**Published:** 2026-06-12

**Authors:** Lisa R. McTaggart, AliReza Eshaghi, Kirby Cronin, Samir N. Patel, Julianne V. Kus

**Affiliations:** 1 Public Health Ontario 153300, Toronto, Ontario, Canada; 2 Department of Laboratory Medicine and Pathobiology, University of Toronto 7938, Toronto, Ontario, Canada; Children's Hospital Los Angeles, Los Angeles, California, USA

**Keywords:** *Mycoplasma pneumoniae*, outbreak, macrolide resistance, *p1 *genotyping

## Abstract

*Mycoplasma pneumoniae* (Mpn) is a common respiratory tract pathogen known to cause cyclic epidemics every 3–4 years. For 4 years (2020–2023), a low positivity rate (0.64%) for Mpn laboratory testing at Public Health Ontario suggested minimal circulation of Mpn in Ontario, Canada, likely owing, in part, to COVID-19-related non-pharmaceutical interventions. In 2024, we observed a dramatic re-emergence of Mpn, characterized by large increases in the number of laboratory-confirmed cases (*n* = 1,122) and the positivity rate (19.1%) of laboratory detections. Patient age distribution of Mpn infections in 2024 did not differ from 2018 to 2019; however, analysis of patient setting at the time of specimen collection as a proxy for disease severity saw a significant increase in the proportion of Mpn-positive specimens collected from patients in hospital emergency departments (ED) (*P* < 0.001), and a decrease in the proportion from patients in inpatient wards (*P* = 0.001). Macrolide-resistant rates were statistically equivalent in 2024 (14.6%) compared to 2018–2019 (12.4%), as was the ratio of *p1*-1 and *p1*-2 adhesin subtypes. Combined 2018–2024 data show that a significantly greater proportion of Mpn-positive pediatric specimens occurred in community clinics/private practices (*P* = 0.001), compared with adults, where a greater proportion were in EDs (*P* = 0.012), or intensive/critical care units (*P* < 0.001). Our findings suggest that the magnitude of Mpn infections in 2024 was not due to a novel strain, but rather due to the increased transmission of strains previously circulating in Ontario following the cessation of COVID-19 pandemic-related non-pharmaceutical interventions, and exacerbated by population immunity debt and the cyclic nature of Mpn.

**IMPORTANCE:**

*Mycoplasma pneumoniae* (Mpn) is a common cause of community-acquired respiratory infection and is known for causing “atypical” pneumonia. Infections show seasonal and cyclical patterns with larger epidemics every 3–4 years. Because Mpn is transmitted via respiratory droplets, incidence was very low during the COVID-19 pandemic when masking and physical distancing were widely utilized. In late 2023 through 2024, many jurisdictions worldwide reported a surge in Mpn cases. This increase, along with reports of emerging resistance to first-line treatment macrolide antibiotics, raised concerns about the management of severe infections. Here, we describe the post-COVID-19 pandemic re-emergence in Mpn in Ontario, Canada. Despite high case numbers detected in 2024, infections were not more severe than in pre-pandemic years (2018–2019). Mpn genotype ratios and macrolide resistance rates were statistically comparable between 2018/2019 and 2024, suggesting re-emergence of previously circulating strains rather than the introduction of a novel hypervirulent strain.

## INTRODUCTION

*Mycoplasma pneumoniae* (Mpn) is a common cause of upper respiratory tract infections and is a major cause of community-acquired pneumonia (CAP) among the pediatric and adult populations (1). Although Mpn infections are often self-limiting, hospitalization can be required to treat severe disease, such as severe pneumonia, encephalitis, and extrapulmonary complications. Most infections occur in school-aged children and adolescents; however, adults with underlying health conditions are at high risk for severe complications (1). Mpn lack a cell wall, rendering beta-lactam antibiotics, common first-line antibiotics for CAP, ineffective. Macrolides and tetracyclines, which target ribosomes involved in protein synthesis, are the first-line therapy for Mpn infections, followed by DNA replication-disrupting fluoroquinolones as a second-line choice (1). Failure to suspect Mpn upon initial presentation commonly results in delays of appropriate treatment.

Mpn infections cause periodic epidemics, typically every 3–4 years (2, 3). However, the non-pharmaceutical interventions (NPIs), such as universal masking, physical distancing, and travel restrictions, introduced in many countries to stem the transmission of severe acute respiratory syndrome coronavirus 2 (SARS-CoV-2), also interrupted the typical cyclical fluctuations in Mpn cases, such that very few Mpn infections were noted during the coronavirus disease 2019 (COVID-19) pandemic years (2020–2022) (4, 5). Then, in late 2023, a surge in Mpn cases was reported in many regions of the world, including Asia, Oceania, the Americas, and Europe (6–15). The unprecedented high number of infections created challenges for the healthcare community in responding to the increase in patients requiring advanced treatment and raised questions concerning the possible spread of a hypervirulent strain or macrolide-resistant Mpn (MRMP) (6). Although MRMP rates remain moderate or low in many regions, it has become a significant concern in Asia, with very high rates of MRMP reported in China (99.1%) and South Korea (78.5%) (11, 16, 17).

Due to challenges with traditional minimum inhibitory concentration testing of Mpn, antimicrobial resistance is often predicted using genetic analysis for identification of resistance markers *in silico*; sequence analysis can also be used for strain typing and tracking for epidemiological investigations. Resistance to macrolides is primarily caused by nucleotide substitutions at domain II and/or V of the 23S rRNA gene, causing alterations in the macrolide binding site. Mutations at nucleotide 2063 (A2063G/C/T), 2064 (A2064G/T), 2067 (A2067G), and 2617 (C2617G) have been shown to be associated with increased MICs to macrolides (1, 18). Nonsynonymous substitutions causing mutations in L4 and L22 ribosomal proteins or in GyrA and ParC may also confer resistance to macrolides or quinolones, respectively. While these genotype-phenotype relationships are not well established for Mpn (18), evidence from a related species, *Mycoplasma genitalium,* suggests a high correlation between fluoroquinolone resistance and specific amino acid substitutions in quinolone resistance-determining regions (QRDRs), namely M95 or D99 of GyrA and S83 or D87 of ParC (19, 20). One method of strain typing involves sequencing the *p1* adhesin gene, the major external surface protein of Mpn, which facilitates cytoadherence crucial for colonization of the respiratory tract and disease progression (1, 21). As a cell surface protein, P1 also serves as an immunodominant protein, triggering the immune response during infection. As such, antigenic variation of P1 facilitated by homologous recombination of the two repetitive elements (*RepMP2/3* and *RepMP4*) helps Mpn evade the host immune system and acts as a target for strain typing for epidemiological purposes (1). Mpn strains are classified into two major genetic groups, *p1* subtype 1 (*p1*-1) and P1 subtype 2 (*p1*-2), with further categorization into many variants (22).

In this study, we use provincial public health laboratory data to describe the fluctuation in case counts and positivity rates of molecular detection of Mpn in Ontario, Canada’s most populous province, from 2018 to 2024, encompassing both pre- and post-COVID-19 pandemic years. In response to a large increase in the number of Mpn cases and the positivity rate of detection in 2024 in Ontario, we investigated pre- versus post-pandemic alterations in patient age or patient setting at the time of specimen collection. We used patient setting as a proxy for disease severity based on the assumption that the patient setting at the time of specimen collection provides an indication of the level of medical care required, which directly correlates with the seriousness of the patient’s condition. Additionally, we catalog the *p1* genotypes and prevalence of mutations conferring resistance to macrolides to further characterize the dynamics of Mpn infections in Ontario before, during, and after the COVID-19 pandemic.

## MATERIALS AND METHODS

Public Health Ontario’s laboratory (PHOL) is the provincial microbiology reference laboratory in Ontario, Canada. PHOL performs a large proportion of Mpn testing in the province, receiving samples from community clinics, private medical practices, and many hospitals that do not have in-house testing, thus supporting a wide range of patients and settings across Ontario. To investigate the epidemiology of Mpn infections in Ontario, we collated laboratory data on 1,385 cases represented by positive specimens submitted to PHOL for Mpn qPCR testing between 1 January 2018 and 31 December 2024. Briefly, specimen DNA was extracted using EMAG (bioMérieux, Marcy-l’Etoile, France). Mpn was detected using a validated, in-house developed qPCR assay on a QuantStudio 5 using published primers and probes (23, 24) and TaqMan multiplex master mix (ThermoFisher Scientific, Waltham, MA, USA). For qPCR positive specimens, patient age range and patient setting (community, emergency department [ED], inpatient [non-ICU] ward, intensive care unit/critical care unit [ICU/CCU], or unknown) at the time of specimen collection were recorded. In order to eliminate duplicate specimens from the same patient from the data set using a consistent approach, we retained unique cases associated with the most severe patient setting (ICU/CCU > inpatient [non-ICU] > ED > community) for that patient.

For additional genotypic testing of Mpn qPCR positive specimens, 500 µL of primary respiratory specimen (nasopharyngeal or throat swab in Universal Transport Medium) was inoculated into a dual-phase (broth/agar) flask containing Mpn culture media (Mycoplasma ATCC medium 247). Flasks were incubated aerobically for up to 4 weeks at 35°C–37°C. Growth was monitored by a pH indicated color shift (red to yellow) in the broth. Following microscopic observation of typical colonial morphology (“strawberry-like” appearance), DNA was isolated from 250 µL of this enrichment broth using EMAG (bioMérieux, Marcy-l’Etoile, France) for downstream PCR and amplicon next-generation sequencing (NGS).

PCR amplification was done in singleplex [for *p1* adhesion gene (*mgpA*) and *parC*] or duplex [for 23S/*gyrA* and L4 (*rplD*)/L22 (*rplV*) ribosomal genes]. Pooled amplicons were sequenced by Illumina NGS. Briefly, the targets were amplified using primers (Table 1) and Q5 High-Fidelity 2X Master Mix (New England Biolabs, Ipswich, MA). Amplicons were pooled (combined into a single library preparation) in equimolar ratios (each target was present at the same molar concentration to ensure uniform sequencing coverage) and purified using a 1× ratio of Ampure XP beads (Beckman Coulter, Inc., Brea, USA). Samples were prepared for NGS using the Nextera XT kit (Illumina, San Diego, CA) according to the manufacturer’s instructions. The normalized libraries (12 pM) were pooled and loaded onto a MiSeq v2 reagent cartridge (300 cycles) for sequencing on the Illumina MiSeq system.

**TABLE 1:** Primers used for gene target amplification^*a*^

Gene	Primer	Primer sequence (5′−3′)	Base position	Length	Reference
*23S rRNA*	Mp-23S-F	GGACAACAGGTTAATATTCCTG	121,443	1,498	(25)
	Mp-23S-R	CAATAAGTCCTCGAGCAATTAG	122,940		
*p1 operon 1* (*mpgA*)	Mpn-P1-17F	GCGGGGGTCATATTCAGG	17	4,947	This study This study
	Mpn-P1-4963R	CTGGCTTTGGTGGTACTGGT	4,963		
*gyrA*	gyrA-F	TGGAGTATGCGATGTCGGT	4,845	2,335	(18)
	gyrA-R	ACAGTCTCTAAGCGTTCCT	7,179		(18)
*parC*	parC-F	GCGTTATTACTCCAGGCCAT	158,274	464	(18)
	parC-R	GCTTCCGTATAGCGCATCGC	157,737		This study
*L4 (rplD*)	MNL4F	AAAAGCAGCACCAGTTGTAG	217,008	723	(18)
	MNL4R	GGTTAGAACTGGTTTTAGCA	217,729		(18)
*L22 (rplV*)	*MNL22F*	GTACATAACGGCAAGACCTT	219,417	628	(18)
	MNL22R	GCAAGCCGTTGGAGTTTACT	220,043		(18)

^*a*^ The base position refers to the location in the *M. pneumoniae* M129 genome (NC_000912.1) reference genome.

For *p1* adhesin gene (*mpgA*) analysis, raw sequences were assembled using SKESA (26). Resulting contigs were queried using BLASTn against a *p1* database containing type 1, 1a, 1b, 2, 2a, 2b, 2c, 2c2, 2d, 2f, 2g, and 2j variant sequences (22). The *p1* type was assigned based on the top hit (>99% sequence identity). A neighbor-joining phylogenetic tree of *p1* sequences from 30 isolates selected from Kenri et al. (22) and from this study to demonstrate the relationship between known *p1* types and a novel *p1* type discovered in this study was constructed using the neighbor-joining method in MEGA11 (27).

CLC Genomic Workstation software v8.5.3 (CLC bio, Qiagen) was used for downstream analysis of antimicrobial resistance (AMR) determinants. This included macrolide-resistance markers in the 23S rRNA gene, and genes for L4 (*rplD*) and L22 (*rplV*) ribosomal proteins, as well as fluoroquinolone-resistance markers in *gyrA* and *parC*. Raw fastq were assembled to the reference *M. pneumoniae* M129 genome (NC_000912.1) for *in silico* mutation analysis. Due to constraints on the availability and quality of sample specimens for research purposes or unsuccessful Mpn culture, only samples for 973 of the 1,385 Mpn cases were available for genomic analysis. Due to failed PCR amplification or sequencing, mutation analysis was conducted for between 919 and 966 samples, depending on the target.

Statistical comparisons were conducted using Pearson’s chi-square tests, pairwise chi-square goodness-of-fit tests comparisons, and chi-square post hoc tests using the R packages rstatix and chisq.posthoc.tests and the false discovery rate method to adjust *P* values for multiple comparisons. Fisher’s exact tests from the R stats package were used when expected cell counts were less than 5.

This investigation was conducted as part of Public Health Ontario’s legislated mandate to provide scientific and technical advice as well as operational support in emergency or outbreak situations (28). As this work is considered public health practice and not research, research ethics approval was not required. Specimens and associated data were anonymized prior to use by a PHOL data custodian, and accordingly, individual consent was not required for the secondary use of non-identifiable specimens and associated information.

## RESULTS

### Mpn laboratory testing, 2018–2024

**Fig 1:**
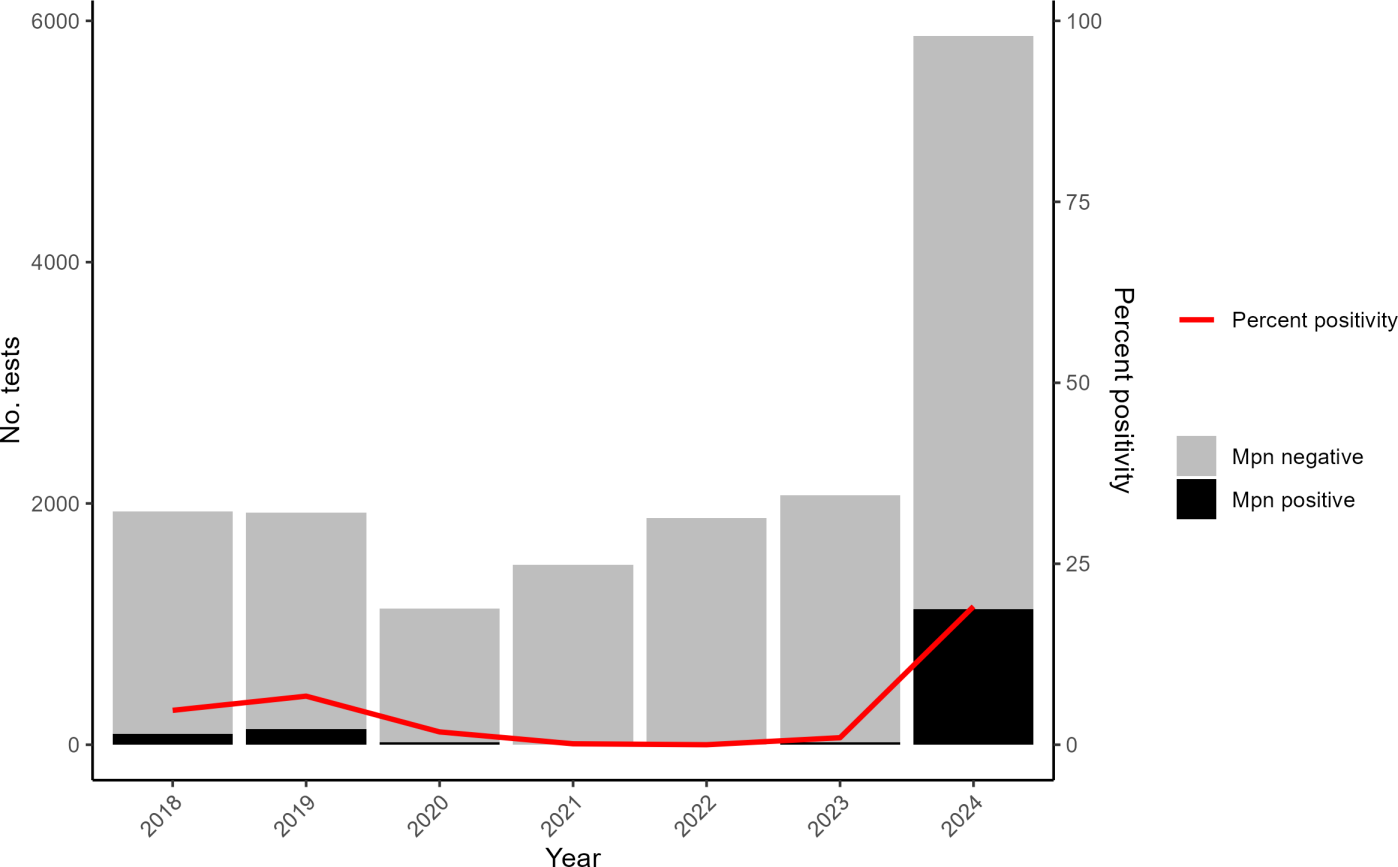
The number of positive and negative Mpn tests and the percentage of positive samples of those submitted to PHOL for Mpn testing, 2018–2024.

The number of cases of Mpn detected by laboratory testing at PHOL fluctuated between 2018 and 2024. An average of 110.5 cases per year were detected in 2018 and 2019 (pre-COVID-19 pandemic years) with a positivity rate of 5.7%. Despite a 41.4% and 22.7% reduction in the number of specimens received for testing in 2020 and 2021, respectively, there was an 80%–100% reduction in cases of Mpn with ≤20 detected each year from 2020 to 2023 and a positivity rate of 0.64% (Table 2; Fig. 1). In 2024 (post-COVID-19 pandemic), both the number of tests (*n* = 5,875) and the number of laboratory-confirmed positive cases (*n* = 1,122) increased compared to previous years, with the percent positivity in 2024 (19.1%) significantly greater than 2018/2019 combined (5.7%) (*P* < 0.001) (Table 2; Fig. 1).

**TABLE 2:** Laboratory cases (*n* = 1,385) of Mpn in Ontario from 2018 to 2024, categorized by patient age, patient setting, macrolide susceptibility (resistance inferred by 23S A2063C, A2063G, A2063T, or A2064G), *p1*-type^*a*^

	2018	2019	2020	2021	2022	2023	2024	*P* value^*a*^
No. of tests	1,936	1,923	1,130	1,491	1,878	2,066	5,875	
No. of positive tests	92	129	20	2	0	20	1,122	<0.001
Percent positivity (%)	4.8	6.7	1.8	0.1	0.0	1.0	19.1	
Patient age (years)								
<1	1	0	0	0	0	0	11	
1–4	11	21	2	0	0	1	170	
5–11	50	60	5	0	0	5	466	0.276
12–17	17	24	8	0	0	5	253	
18–39	7	17	4	0	0	5	112	
40–59	2	6	1	1	0	1	81	
60+	4	1	0	1	0	3	28	
Unknown	0	0	0	0	0	0	1	
Patient setting								
Community	19	18	1	0	0	2	168	
ED	9	4	2	0	0	3	215	
ICU/CCU	0	0	0	0	0	1	24	<0.001
Inpatient	19	56	11	0	0	5	298	
Unknown	45	51	6	2	0	9	417	
Macrolide susceptibility								
Macrolide-sensitive (MSMP)	64	99	12	2	0	8	639	1.000
Macrolide-resistant (MRMP)	5	18	3	0	0	6	110	
Nd	23	12	5	0	0	6	373	
Percent MRMP (%)	7.2	15.4	20.0	0.0	n/a	42.9	14.6	
p1 Type								
Type 1	19	45	4	0	0	8	493	
Type 2	3	5	1	0	0	0	4	
Type 2b	0	0	0	0	0	0	10	<0.001
Type 2c	9	17	4	1	0	1	66	
Type 2j	0	0	0	0	0	3	158	
Type 2novel	0	0	0	0	0	0	5	
Nd	61	62	11	1	0	8	386	

^*a*^ For each category, *P* values < 0.05 denote a statistically significant difference in case distribution by chi-square analysis (excluding unknown, not determined [nd]) between pre-COVID-19 pandemic (2018/2019 combined data) compared to post-COVID-19 pandemic (2024 data).

### Patient age, setting, macrolide susceptibility, and *p1*-subtyping of cases of laboratory-confirmed Mpn, 2018–2024

To better understand the temporal trends in Mpn infections, we analyzed additional patient and laboratory data associated with positive specimens, comparing 2024 data with 2018/2019 (pre-COVID-19 pandemic) combined data. While there was no significant change in patient age distribution (*P* = 0.276) (Table 2), there was a statistically significant change in patient setting where Mpn-positive specimens were collected for testing (*P* < 0.001). Excluding cases where patient setting was unknown, there was an increase in the proportion of cases where Mpn-positive specimens were collected from ED patients (*P* < 0.001) and a decrease in the proportion of specimens from people in inpatient (non-ICU) wards (*P* = 0.001) in 2024 compared to 2018/2019 (Table 2). When stratifying the data to distinguish pediatric patients (<18 years old) and adults (18+), the change in patient setting between 2018/2019 and 2024 was due to a statistically significant increase in pediatric patients having laboratory testing for Mpn ordered while in the ED (0.007), a decrease in pediatric specimens from inpatient (non-ICU) settings (*P* = 0.002), and a statistically significant increase in adults having Mpn testing orders while presenting to the ED (*P* = 0.049).

The percentage of cases with MRMP isolates possessing a 23S A2063C, A2063G, A2063T, or A2064G mutation varied between 2018 and 2024 (range 0% to 40%); however, the resistance rates for 2024 (14.6%) were comparable to 2018/2019 (12.4%) (*P* = 1.000) (Table 2; Table S1).

**Fig 2:**
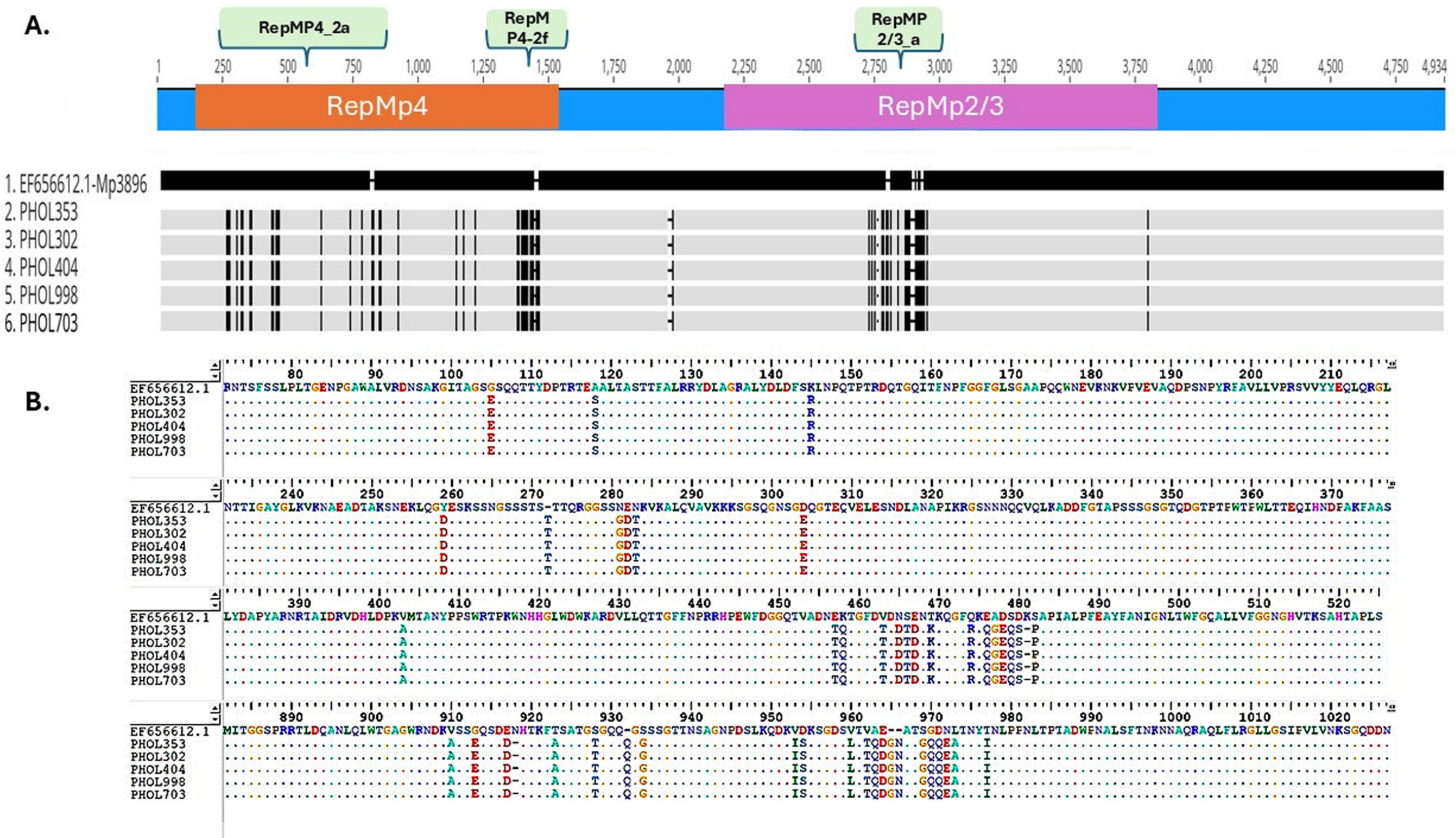
Alignment of novel *p1*-2 variant to closest variant *p1*-2d (EF656612.1-Mp3896). (**A**) A schematic illustration of the nucleotide alignment of *mpn141* encoding a novel *p1*-2 variant from five study samples (PHOL353, PHOL302, PHOL404, PHOL998, and PHOL703) to the most similar *p1*-2 variant, *p1*-2d (EF656612.1). Areas of dissimilarity are represented by black bars. The orange and pink bars indicate the locations of the RepMP4 and RepMP2/3 elements. Regions with higher similarity to *p1*-2a and *p1*-2f than *p1*-2d are indicated. (**B**) Select regions of the amino acid sequence alignment of P1. The conserved amino acids identical to P1-2d are shown in dots. The novel variant amino acids different from the P1-2d are shown in multi-colored letters.

While the proportion of Mpn *p1*-1 and *p1-2* subtypes was statistically equivalent between 2018/2019 (ratio 64:34) and 2024 (ratio 493:243) (*P* = 0.741) (Table 2; Table S1). All p*1*-1 Mpn were classical p*1*-1; neither p*1*-1a nor p*1*-1b was observed. However, there was a small but statistically significant change in the distribution of *p1*-2 variants with a decrease in the proportion of classical *p1*-2 (*P* < 0.001) and increases in *p1*-2c (*P* < 0.001) and *p1*-2j (*P* < 0.001), as well as the detection of five cases with a novel *p1*-2 variant (GenBank accession numbers PX960614–PX960618) that was phylogenetically distinct from other *p1*-2 variants (Table 2; Fig. S1). The novel variant identified here was most similar to *p1*-2d (EF656612.1). However, select sub-regions of *RepMP4* and *RepMP2/3* showed greater *mgpA* (*mpn141*) nucleotide and corresponding P1 amino acid sequence similarities to variants 2a and 2f than 2d, suggesting homologous recombination between the *p1*-type 2 variants (Fig. 2).

### Patient setting and Mpn macrolide susceptibility in pediatric patients and adults

**Fig 3:**
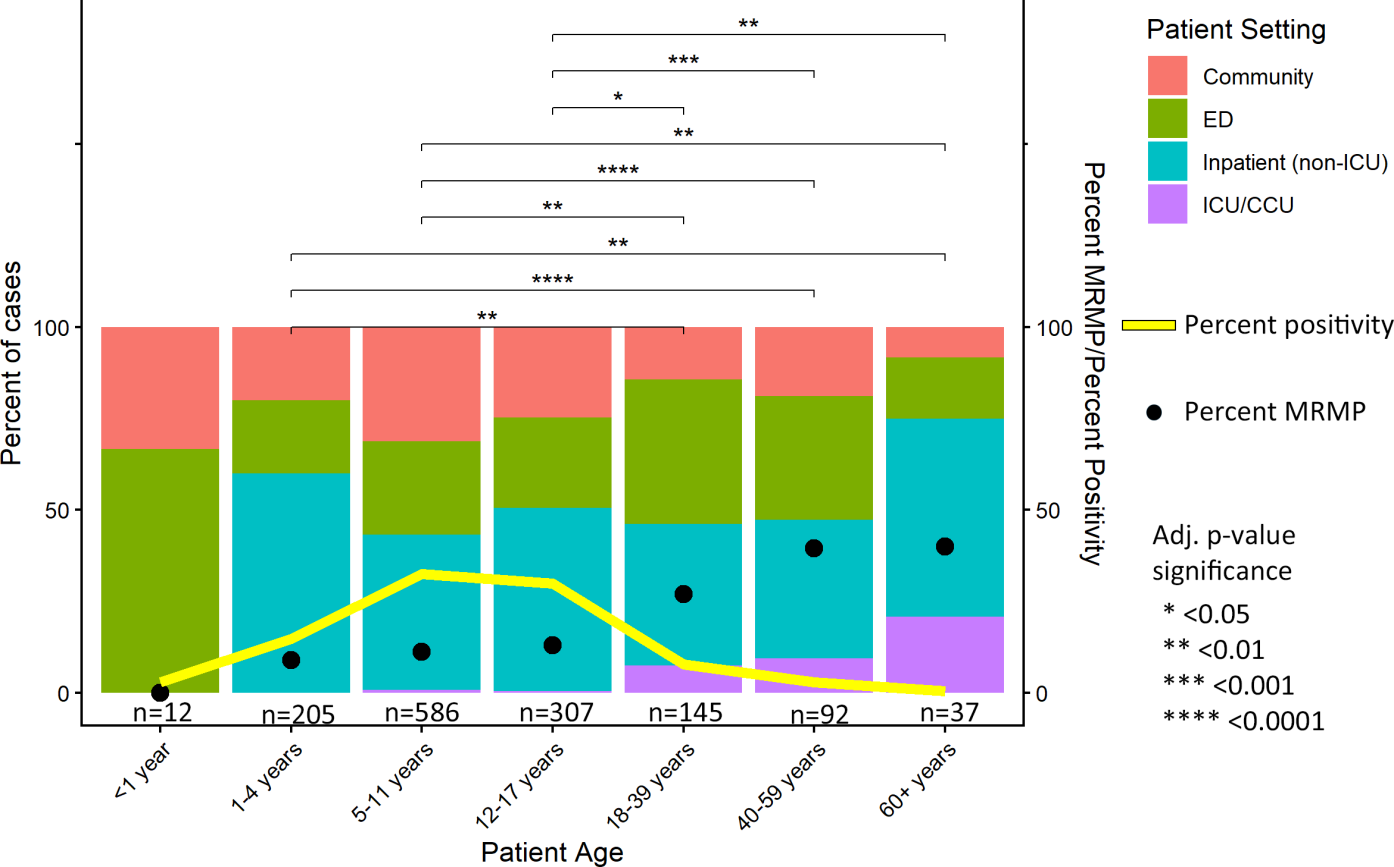
Distribution of Mpn cases (2018–2024) detected when patients presented in community or hospital (ED, inpatient [non-ICU], ICU/CCU) settings as a proxy for disease severity based on patient age. Percent positivity (yellow line) and percent MRMP (black dots) for each age group are shown together with statistically significant comparisons (*P* < 0.05) of the proportion of MRMP samples between age groups.* *P*-value < 0.05, ** *P*-value < 0.01, *** *P*-value < 0.001, **** *P*-value < 0.0001.

Combining all data from 1,385 cases from 2018 to 2024, variations in patient setting at the time of specimen collection for Mpn testing and macrolide resistance rates were evident when comparing pediatric patients (<18 years old) and adults (18+). Pediatric patients represented the vast majority of cases (1,110/1,384, 80.2% versus adults, 274/1,384, 19.8%), with 5–11-year-olds (586/1,384, 42.3%) exhibiting the most cases. The percent positivity rates were highest for 5–11-year-olds (32.5%) and 12–17-year-olds (29.9%), which were statistically higher than adults aged 18–29 (7.9%), 40–59 (3.0%), and 60+ (0.5%) (*P* < 0.001). The proportion of pediatric patients and adults presenting in different patient settings (community, ED, ICU/CCU, inpatient [non-ICU]) at the time of specimen collection for laboratory testing was significantly different (*P* < 0.001). A greater proportion of Mpn-positive specimens was collected from adults while in the ED (76/217, 35.0%, *P* = 0.012) or ICU/CCU (21/217, 9.7%, *P* < 0.001) compared to pediatric patients (157/637, 24.6%; 4/637, 0.6%, respectively). A greater proportion of Mpn-positive pediatric specimens were received from community clinics (175/637, 27.5%) compared to specimens from adults (33/217, 15.2%) (*P* = 0.001) (Fig. 3). While the overall rate of MRMP was 14.7%, a significantly greater proportion of Mpn samples from adults from all age groups (18–39 years, 40–59 years, 60+ years) was MRMP (23/85, 27.1%; 19/48, 39.6%; 8/20, 40.0%, respectively) compared to pediatric age groups 1–4 years (14/156, 9.0%), 5–11 years (49/434, 11.3%), and 12–17 years (29/224, 12.9%) (Fig. 3).

**Fig 4:**
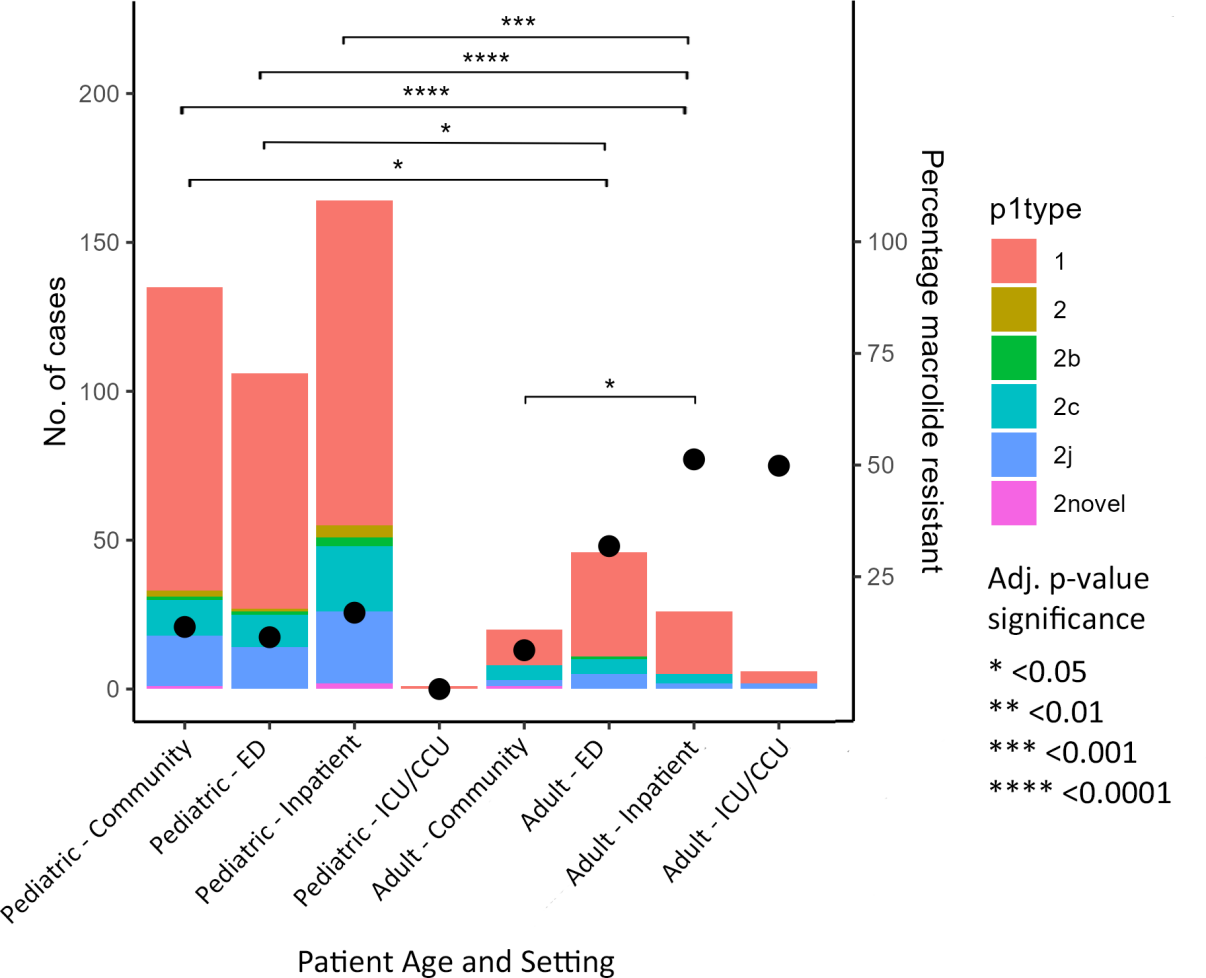
Distribution of variants of *p1* subtypes and macrolide resistance rates (black dots) among cases of Mpn (2018–2024) in pediatric patients (<18 years old) or adults (18+) from samples collected in various healthcare settings. Statistical significance (*P* < 0.05) of comparisons of the proportion of MRMP samples is shown. * *P*-value < 0.05, ** *P*-value < 0.01, *** *P*-value < 0.001, **** *P*-value < 0.0001.

There was no statistically significant correlation between variants of *p1* subtypes and patient setting (*P* = 0.872) for either pediatric patients (<18 years old) (*P* = 0.9301) or adults (18+) (*P* = 0.432) (Fig. 4). However, the macrolide resistance rates varied depending on age and patient setting. MRMP rates were elevated from samples from adults in the ED (32.0%, 16/50), ICU/CCU (50.0%, 3/6), and inpatient (non-ICU) (51.4%, 18/35) hospital settings. There was no statistically significant difference in macrolide resistance rates for pediatric specimens collected in different patient settings. However, the proportion of MRMP samples from adult Mpn cases in inpatient (non-ICU) wards (18/35, 51.4%) was significantly higher than community clinics (2/23, 8.7%) (*P* = 0.015) (Fig. 4). Furthermore, the proportion of MRMP samples was significantly higher in adults presenting in the ED (16/50, 32.0%) than in pediatric patients presenting in the community (21/151, 13.9%) (*P* = 0.037) or the ED (13/112, 11.6%) (*P* = 0.021) and between adults in inpatient (non-ICU) wards (18/35, 51.4%) compared to pediatric patients submitting specimens for laboratory testing at community clinic visits (21/151, 13.9%) (*P* < 0.001), the ED (13/112, 11.6%) (*P* < 0.001) or from inpatient (non-ICU) settings (33/193, 17.1%) (*P* < 0.001) (Fig. 4).

### *In silico* analysis of mutations in non-23S AMR targets

Finally, single-nucleotide variants (SNVs) in genes encoding proteins targeted by macrolides (ribosomal proteins L4 and L22) and fluoroquinolone resistance-determining regions (QRDR) of ParC and GyrA, resulting in synonymous or nonsynonymous mutations, were tabulated. For the L4 ribosomal protein, 9.3% (88/946) of cases had the K27N mutation, while 34.6% (328/947) had M144V; only SNVs causing synonymous mutations were detected in the *rplV* (L22) gene (1.4% [13/946] had SNV C9T; 65.3% [618/946] had SNV C204T) (Table S1). Similarly, a SNV resulting in a synonymous mutation (SNV C369G) was detected in *parC* of 35.0% (330/944) of isolates, while GyrA mutations D149G, A533S, A581T, and C705G were observed in 13.8% (127/921), 34.2% (315/921), 9.7% (89/920), and 34.2% (314/919) of isolates, respectively (Table S1). Co-mutations of K27N and A581T in L4 and GyrA, respectively, were observed among some strains of P1-type 1. Additionally, signature mutations of M144V and A533S (in L4 and GyrA, respectively) were identified primarily in strains belonging to *p1*-type 2 (Table S1).

## DISCUSSION

For 4 years (2020, 2021, 2022, 2023), Mpn testing at the provincial public health laboratory in Ontario, Canada, recovered of ≤20 cases of Mpn infections per year, and the positivity rate for 2020–2023 was 0.64%. In 2024, infections spiked to 1,122 cases with a positivity rate of 19.1%. Mpn cases in 2024 surged to a level approximately 10× that observed in 2018–2019 (with 92 and 129 cases per year, respectively), and the positivity rate of 19.1% was significantly higher than pre-COVID-19 pandemic years 2018/2019 (5.7%). This underestimates the total number of cases in Ontario, as the actual positivity rate is unknown since other laboratories also perform some Mpn testing. Our data, along with other data (29), suggest widespread community transmission in 2024. Many global regions, including jurisdictions in Europe, Asia, the Americas, and Oceania, reported a similar post-COVID-19 pandemic re-emergence in Mpn infections starting in late 2023 following a dearth of cases in 2020–2022. The re-emergence of Mpn infections in 2023 was identified as an epidemic characterized by both a high number of detections and an elevated detection rate (6–15, 29, 30). In Ohio, USA, an area with regional proximity to Ontario, Canada, a similar trend in Mpn cases was noted with a sharp increase starting in the summer of 2024 (30, 31).

The low number of cases observed globally in 2020–2023 was likely due in part to NPIs aimed at reducing SARS-CoV-2 transmission, which also reduced the transmission of other respiratory pathogens, such as Mpn (4, 5). While the reduction in Mpn cases following NPIs implementation was predictable (5), the delay in re-emergence of Mpn after NPIs were lifted was somewhat unexpected (32). Genotyping studies show that strains from the 2023 re-emergence of Mpn were caused by multiple genotypes circulating regionally in the years prior (8, 15, 33). Coupled with the observation that region-specific MRMP rates remained unchanged, these findings suggest that the global re-emergence of Mpn starting in 2023 was caused by increased transmission of endemic strains and not the circulation of a novel, hyper-virulent strain. Similarly, we conclude that the increase in Mpn cases in Ontario in 2024 was due to the re-emergence of strains circulating in Ontario in previous years, since the MRMP rates, as well as the ratio of *p1*-1 and *p1*-2 genotypes, did not differ between 2018/2019 and 2024. Recent modeling of data from 65 sites in 29 countries suggested that the delayed re-emergence of Mpn in 2023–2024 was related to the dramatic (90%) reduction in transmission during the COVID-19 pandemic, coupled with the prolonged generation time due to a long incubation period (3 weeks) (6). Population immunity debt, which is an increase in the number of susceptible individuals in a population caused by a prolonged period where there was a lack of natural exposure due to COVID-19 pandemic NPIs, may have contributed to the magnitude of cases detected during re-emergence (7, 34).

Despite the spike in Mpn cases in 2024, analysis of patient settings at the time of specimen collection for laboratory testing as a proxy of disease severity suggests that Mpn infections were not more severe in 2024 compared to 2018/2019. Mpn-positive specimens collected during hospital ED visits increased, but the number of cases diagnosed in non-ICU ward inpatients decreased. We considered inpatient (non-ICU) wards as representative of more severe patient symptoms since they are used to manage more complex, high-acuity patients requiring specialized, long-term treatment. By contrast, while specimen collection for Mpn from patients in the ED would include some high-acuity patients, it would also include many non-critical patients who were not admitted to the hospital. If an ED patient was admitted to the hospital (inpatient or ICU/CCU) and a second specimen was submitted for testing, we would have tabulated the data from the inpatient or ICU/CCU specimen. However, this insight is limited since we lacked access to additional patient information, including co-morbidities, treatment, and disease course/outcome. Most studies evaluating hospitalization or ICU admissions during Mpn infections reported no change in these statistics between pre-COVID-19 pandemic and re-emergence time periods (6, 13), and there was no statistical difference in the number of deaths (6). Likewise, clinical characteristics and patient outcomes were similar during and after the COVID-19 pandemic, with the exception of increased likelihood of developing fever for post-COVID-19 pandemic infections (35). Although disease severity appears statistically unchanged, the increase in case volume resulted in pressure on healthcare delivery systems. Additionally, it is important to note that high numbers of cases during large-scale surges statistically make it more likely to encounter rare but severe extrapulmonary complications, such as encephalitis and stroke (36).

Combining data from 2018 to 2024, a greater proportion of Mpn-positive pediatric specimens were received during visits to community clinics than adult specimens; a greater proportion of adults submitted Mpn-positive specimens from a hospital setting, specifically ED or ICC/CCU, compared to pediatric patients. Although Mpn infections often result in mild, self-resolving symptoms, they can lead to severe community-acquired pneumonia requiring hospitalization, the incidence of which increases with age (37–40). Interestingly, the MRMP rates were higher among positive Mpn samples from adults of all age groups (18–39, 40–59, 60+ years old) compared to pediatric patients (1–4, 5–11, 12–17 years old). While we assume that the same proportion of MRMP and macrolide-sensitive Mpn (MSMP) strains circulate among pediatric patients and adults, a greater proportion of adults may require hospital-based care, especially in cases of infections with MRMP, possibly when prior empiric macrolide treatment fails to resolve the infection. While initial clinical presentations of MRMP are often similar to MSMP, macrolide resistance is associated with a more protracted clinical course. This includes significantly longer febrile periods, prolonged hospitalization, longer duration of antibiotic treatment, and longer defervescence time after macrolide treatment, reflecting increased clinical morbidity due to reduced treatment efficacy (41).

Genotyping of the *p1* adhesin gene of Mpn is frequently used for strain tracking, to predict disease severity, including macrolide resistance, and to understand pathogen evolution (21). Yearly estimates attributed approximately two-thirds of Mpn cases in Ontario to *p1*-1. Similar to other North American jurisdictions, *p1*-1a and *p1*-1b variants were not detected (42). Of the remaining cases, *p1*-2c and *p1*-2j were the predominant subtype *p1*-2 variants in 2024, and a novel *p1*-2 sequence was identified. Historically, *p1*-1 was the dominant subtype, and although it remains the predominant subtype in many regions such as China (8, 10, 11), subtype *p1*-2 variants have emerged in other areas, notably Japan (*p1*-2c and *p1*-2j) (22, 43), and the US (42). While some studies have noted a high prevalence of macrolide resistance within specific *p1*-1 populations, the association between P1 subtype, antimicrobial resistance, and disease severity remains a subject of ongoing clinical debate and may vary significantly by geographic region (40–43). Because it contains multiple immunogenic epitopes, the P1 protein is thought to undergo continual incremental evolution. Previous studies suggest that homologous DNA recombination between repetitive elements generates novel *p1* variants (1, 21), such as the one noted in this study. This mechanism creates antigenic variation, which facilitates evasion of the host’s immune system, potentially allowing for repeated infections. Future work involving MLST may provide additional insight into circulating Mpn strains and help identify potential sources of recombination that contributed to the evolution of the novel *p1*-2 variant.

Due to a lack of a cell wall, Mpn lacks susceptibility to beta-lactam antibiotics, making macrolides the preferred treatment option, with fluoroquinolones as alternatives (1, 44). For 2018–2024, macrolide resistance was predicted in 14.7% of Ontario isolates based on *in silico* identification of point mutations A2063C, A2063T, A2063G, and A2064G in the V domain of the 23S ribosomal RNA within the large (50S) ribosomal subunit, altering the macrolide binding site (18). Macrolide resistance rates vary widely among different regions globally but have remained regionally consistent in recent years (16). The highest MRMP rates are reported in Western Pacific regions (overall 53.4%) (16), with very high rates in China (99.1%) (11) and South Korea (78.5%) (17), while the lowest rates are reported in Europe (overall 5.1%) (16), with very low rates in Denmark (<2%) (13). In North America, MRMP rates are lower in the United States (5%–10%) (16, 30) than in Canada (11%–20%) (16). Based on PHOL data, macrolide-resistance rates for Mpn in Ontario have remained relatively constant at 12.1% in 2011–2012 (45), 12.4% in 2018–2019, and 14.6% in 2024 and are consistent with an 11.8% MRMP rate observed in a smaller, regional study spanning January 2024 to April 2025 (29). Appropriate antimicrobial stewardship is crucial for suppressing the rise in macrolide resistance rates.

In addition to 23S rRNA, we report mutations in *rplD* and *rplV* encoding ribosomal proteins L4 and L22, respectively, potentially conferring macrolide resistance, as well as g*yrA* and *parC*, which encode DNA gyrase and topoisomerase IV, quinolone targets that are essential for DNA replication and cell division. All *rplV* and *parC* SNVs observed among samples in this study resulted in synonymous amino acid substitutions, which are predicted to have no impact on antimicrobial susceptibility. SNVs observed in *gyrA* encoded nonsynonymous substitutions D149G, A533S, and C705G, which are not located in the QRDR but were previously associated with susceptibility to levofloxacin (18), or else the phenotypic effect on susceptibility is unknown (A581T). Likewise, the K27N and M144V mutations predicted for ribosomal protein L4 (*rplD*) sequenced in this study were previously associated with macrolide (erythromycin, azithromycin, josamycin) susceptibility (18). Therefore, mutation analysis of L4, L22, GyrA, and ParC among Mpn in this study predicts no additional macrolide or quinolone resistance beyond that predicted by 23S sequence analysis.

This study has limitations. The actual positivity rate of Mpn in Ontario is unknown since Mpn testing occurs in other laboratories and hospitals in addition to PHOL. The study does not account for cases diagnosed empirically based on patient symptoms or at other laboratories. Clinical surveillance in this study is limited since Mpn is not included as a Disease of Public Health Significance in Ontario. Study data were limited to what was provided on the specimen testing requisitions; thus, information on clinical severity, disease course/outcome, and/or underlying co-morbidities was not available. Patient settings at the time of specimen collection may not accurately portray the severity of individual cases, and we were unable to track individual patient-level test results over time. Also, we did not investigate co-infections with other respiratory bacteria or viruses, which are known to exacerbate disease severity (11). Antimicrobial susceptibility was determined *in silico* by genetic analysis and was not confirmed by phenotypic testing.

In conclusion, we report a surge in Mpn cases in 2024 in Ontario, a North American jurisdiction, comparable to the delayed post-COVID-19 pandemic re-emergence of Mpn observed in late 2023 in Asia, Europe, the Americas, and Oceania. The data presented in this study are consistent with the hypothesis that the timing and intensity of the surge in Mpn cases is due to a re-emergence of strains circulating prior to the COVID-19 pandemic, influenced by the cessation of pandemic-related NPIs and the ~3 week generation time of Mpn. Additionally, the prevalence of MRMP strains and the susceptibility of older adults to severe Mpn disease warrant attention when considering medical treatment options for Mpn infections.

## Data Availability

The nucleotide sequences of the novel P1 sequence obtained in this study have been deposited in GenBank under accession numbers PX960614 through PX960618.

## SUPPLEMENTAL MATERIAL

Figure S1 jcm.00188-26-s0001.tif Phylogenetic tree by neighbor-joining method of 30 *Mycoplasma pneumoniae* P1 sequences showing 14 different subtype variants, including a novel subtype variant identified in this study. Bootstrap values (n = 200) are indicated next to the nodes. Evolutionary distances were computed by Maximum Composite Likelihood method and are in the units of the number of base substitutions per site. NCBI accession numbers are listed where applicable. All other sequences were derived in this study.

Table S1 jcm.00188-26-s0002.xlsx Genetic analysis of study isolates, including year, p1 subtyping, and mutation analysis of antimicrobial resistance markers.

ASM does not own the copyrights to Supplemental Material that may be linked to, or accessed through, an article. The authors have granted ASM a non-exclusive, world-wide license to publish the Supplemental Material files. Please contact the corresponding author directly for reuse.
