# Supplementary figures and images for: Post-pandemic surge of Mycoplasma pneumoniae in Ontario, 2024: molecular surveillance and resistance trends relative to 2018–2023
Source: J Clin Microbiol. 2026 Jun 12;64(7):e00188-26. doi: 10.1128/jcm.00188-26 (PMC13343838; doi:10.1128/jcm.00188-26)

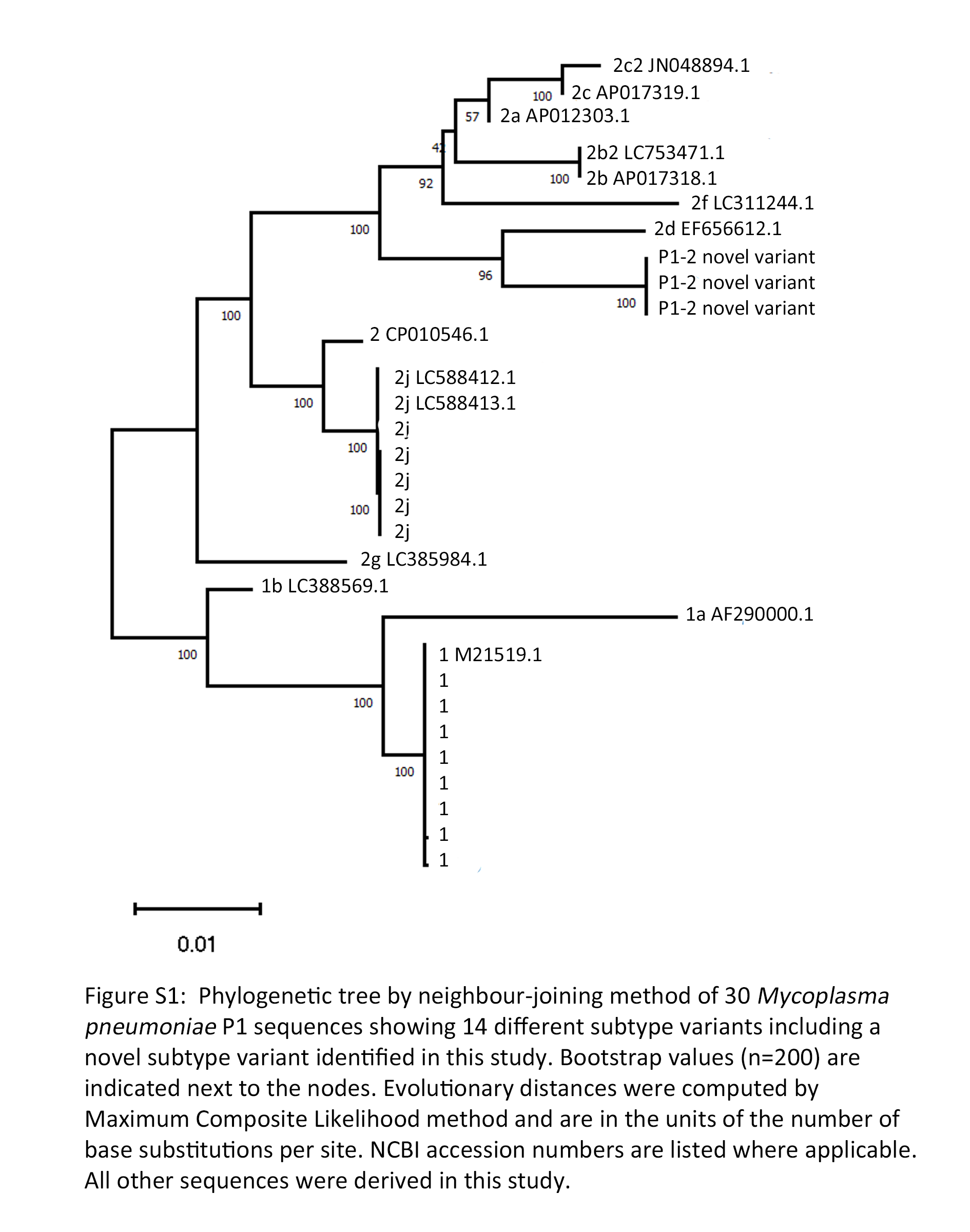

Supplement: Figure S1 — Phylogenetic tree by neighbor-joining method of 30 Mycoplasma pneumoniae P1 sequences showing 14 different subtype variants, including a novel subtype variant identified in this study. Bootstrap values (n = 200) are indicated next to the nodes. Evolutionary distances were computed by Maximum Composite Likelihood method and are in the units of the number of base substitutions per site. NCBI accession numbers are listed where applicable. All other sequences were derived in this study. [file jcm.00188-26-s0001.tif]
